# A Rare Case of Reverse Takotsubo Cardiomyopathy in a 28-Year-Old Female in Peripartum Period

**DOI:** 10.7759/cureus.30504

**Published:** 2022-10-20

**Authors:** Misbahuddin Khaja, Petr Stastka, Sameer Kandhi, Vikram Itare, Amber Latif, Arundhati Dileep

**Affiliations:** 1 Internal Medicine/Pulmonary Critical Care, Icahn School of Medicine at Mount Sinai/BronxCare Health System, Bronx, USA; 2 Medicine, BronxCare Health System, Bronx, USA; 3 Internal Medicine, BronxCare Health System, Bronx, USA; 4 Internal Medicine, American University of the Caribbean School of Medicine, Cupecoy, SXM; 5 Pulmonary, BronxCare Health System, Bronx, USA

**Keywords:** takotsubo cardiomyopathy, 3d echocardiogram, : peripartum cardiomyopathy, acute systolic heart failure, peripartum period

## Abstract

Reverse Takotsubo cardiomyopathy is a rare variant of Takotsubo cardiomyopathy in which ballooning occurs at the base of the heart rather than the apex, which is hyperkinetic. Takotsubo cardiomyopathy is usually considered in postmenopausal women, but a growing number of cases of the reverse variant are emerging in peripartum women. We present a case of peripartum reverse Takotsubo cardiomyopathy in a 23-year-old G1P0 female at 38 weeks gestation, induced by preeclampsia with severe features. An echocardiogram performed in the immediate post-cesarean period demonstrated a severely reduced ejection fraction of 25%, which was improved to 43% over the next 48 hours with diuresis. Therefore, it is imperative to differentiate Takotsubo cardiomyopathy occurring in the peripartum period from peripartum cardiomyopathy, which has a similar clinical presentation, to diagnose this condition and improve patient outcomes.

## Introduction

Takotsubo cardiomyopathy (TCM), also referred to as “broken heart syndrome,” is a rare cardiac syndrome first documented in a 1983 publication in women of Japanese descent [1]. It occurs most commonly in postmenopausal women and is triggered by physical or emotional stress [1]. TCM often mimics acute coronary syndrome (ACS) in its clinical presentation, occurs in women with no previous cardiac issues, and does not demonstrate obstructive coronary artery disease on an angiogram [1, 2]. It is characterized by an acute but often reversible left ventricular (LV) dysfunction with ballooning of the LV apex [2]. Reverse Takotsubo cardiomyopathy (rTCM) is an exceedingly rare variant in which ballooning occurs at the base of the heart rather than the apex, which is instead hyperkinetic [3].

TCM has historically been thought of in the context of postmenopausal women, but a growing number of reported cases are emerging in peripartum women [2]. Because it has been so widely accepted that TCM primarily affects postmenopausal women, it may be the case that a significant number of cases of peripartum Takotsubo cardiomyopathy (PTCM) have been misdiagnosed as peripartum cardiomyopathy (PPCM), which is usually a diagnosis of exclusion [2].

Clinically, it is essential to differentiate peripartum Takotsubo cardiomyopathy (PTCM) from PPCM because the treatment and long-term outcomes vary significantly [2]. Despite LV function being similar at the onset of these two conditions, the prognosis is considerably more favorable in patients with PTCM, with more prompt LV systolic function normalization [2, 4].

The incidence of TCM in patients with elevated troponin is 2% [1, 5]. However, the reports on the incidence of rTCM are variable [1]. An extensive study that analyzed 1,750 patient records from the International Takotsubo Registry found that 2.2% of TCM patients had the reverse variant [1]. Postmenopausal women between the ages of 60-70 represent the majority of TCM cases, while men represent only 10%. Notably, rTCM has been observed chiefly in younger women [1].

The global incidence rate of PPCM is highly variable based on the region. For example, in Nigeria, reports indicate incidence as high as one in 100 deliveries [6]. In contrast, Japan reports an incidence of one in 20,000 deliveries. In the United States, incidence reportedly ranges from one in 1,000 to one in 4,000 and may be increasing due to the rising maternal age and increased multiparous pregnancies due to a surge in the success of modern fertility techniques [6].

The etiology of TCM is not fully understood. Several hypotheses have been proposed, with the most uniformly accepted including microvascular dysfunction, catecholamine-induced cardiotoxicity, and complex neuroendocrine physiology [5]. Proposed mechanisms for rTCM are similar, including estrogen deficiency [1]. The etiology of PPCM also remains poorly understood, but leading theories suggest that genetic predisposition, myocardial inflammation, and vascular dysfunction may play a role [6, 7].

We present a case of reverse peripartum Takotsubo cardiomyopathy (rPTCM) induced by preeclampsia. Expanding the literature on this rare topic is essential in establishing a better understanding of the nuanced diagnosis of this syndrome and differentiating this entity from the diagnosis of exclusion PPCM.

## Case presentation

A 23-year-old G1P0 female at 38 weeks gestation with a previous medical history of class II obesity and hypertension, not pharmacologically managed, presented to the gynecology clinic with the chief medical complaint of high blood pressure of 168/121 mmHg and sudden onset difficulty breathing. The patient had experienced shortness of breath for two days. She endorsed several episodes of nausea and emesis, with the most recent episode occurring the night prior. She denied headaches, blurry vision, slurred speech, epigastric pain, vaginal bleeding, and vaginal discharge. The patient had no significant previous obstetric, gynecologic, surgical, or family history. She denied food and drug allergies. She denied illicit and recreational substance use and alternative and supplemental health practices. She reported a safe relationship and environment.

The patient was hypertensive at 170/120 mmHg. Her temperature was 99.1°F. She was tachycardic at 98 bpm and tachypneic at 29 per min, demonstrating evident dyspnea. During the examination, the patient developed severe respiratory distress; she received 20mg of intravenous labetalol and magnesium sulfate and was immediately taken for C-section. The patient was intubated with an endotracheal tube. The lower segment cesarean section was performed. No blood products were administered. The patient was transferred to the ICU and intubated on the mechanical ventilator from the operating room as there was difficulty weaning her off the ventilator. On auscultation, breath sounds were diminished bilaterally, with diffuse bilateral crackles and fine rales appreciated. Jugular venous distension was not demonstrated. Her lower extremities demonstrated trace edema. The physical examination was otherwise unremarkable. The patient was sedated and started on nitroglycerin drip postpartum and fentanyl drip to facilitate mechanical ventilation with positive end-expiratory pressure of 5 and FiO2 of 40%.

**Figure 1:**
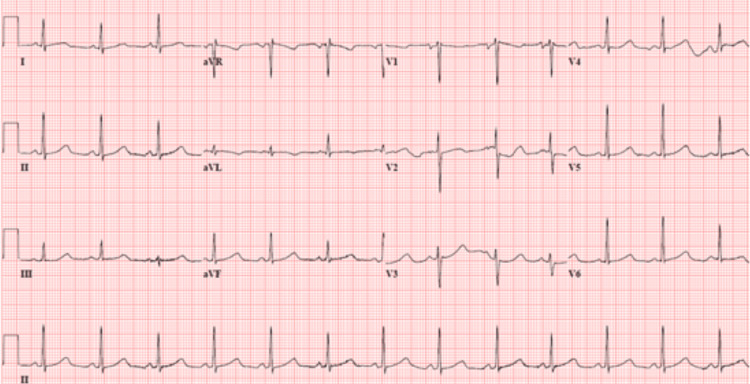
12 Lead Electrocardiogram performed in the preoperative period demonstrating normal sinus rhythm, with no acute changes of ischemia.

**Figure 2:**
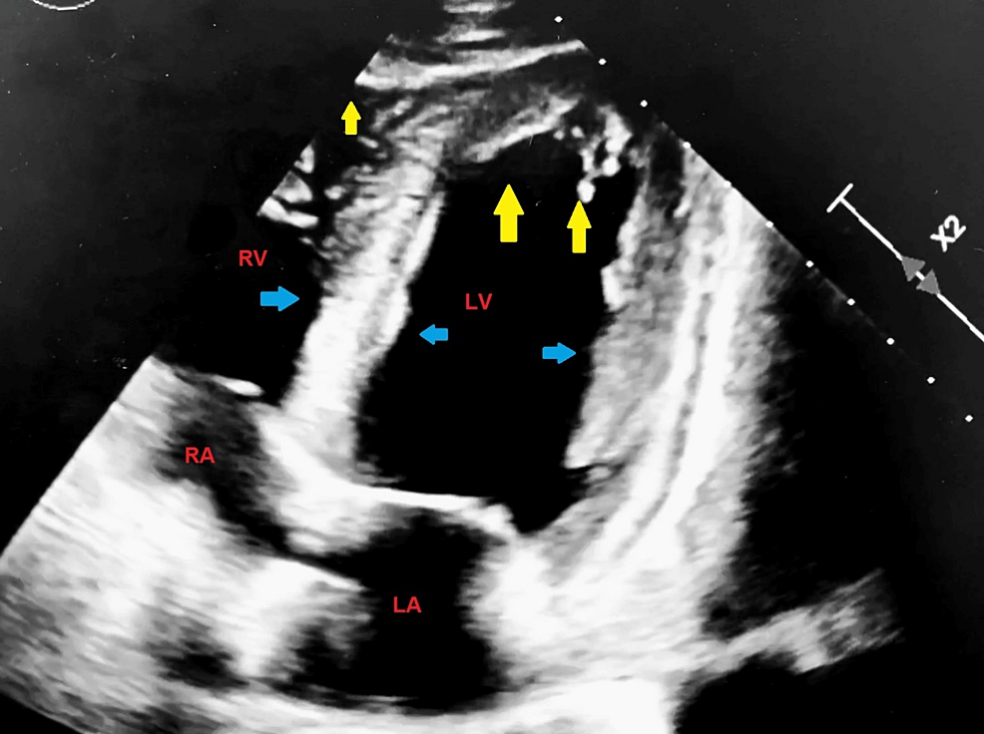
Four Chamber Apical View of 2D Echocardiography performed in the immediate postoperative period demonstrating heart failure with severely reduced ejection fraction (25%). Hyperkinesis of the apices (yellow arrows) of the left and right ventricles was noted with hypokinetic mid-basal segments (blue arrows) which is seen with rTCM. LV: Left Ventricle; LA: Left Atrium; RA: Right Atrium; RV: Right Ventricle; 2D: Two Dimensional; rTCM: Reverse Takotsubo Cardiomyopathy

**Figure 3:**
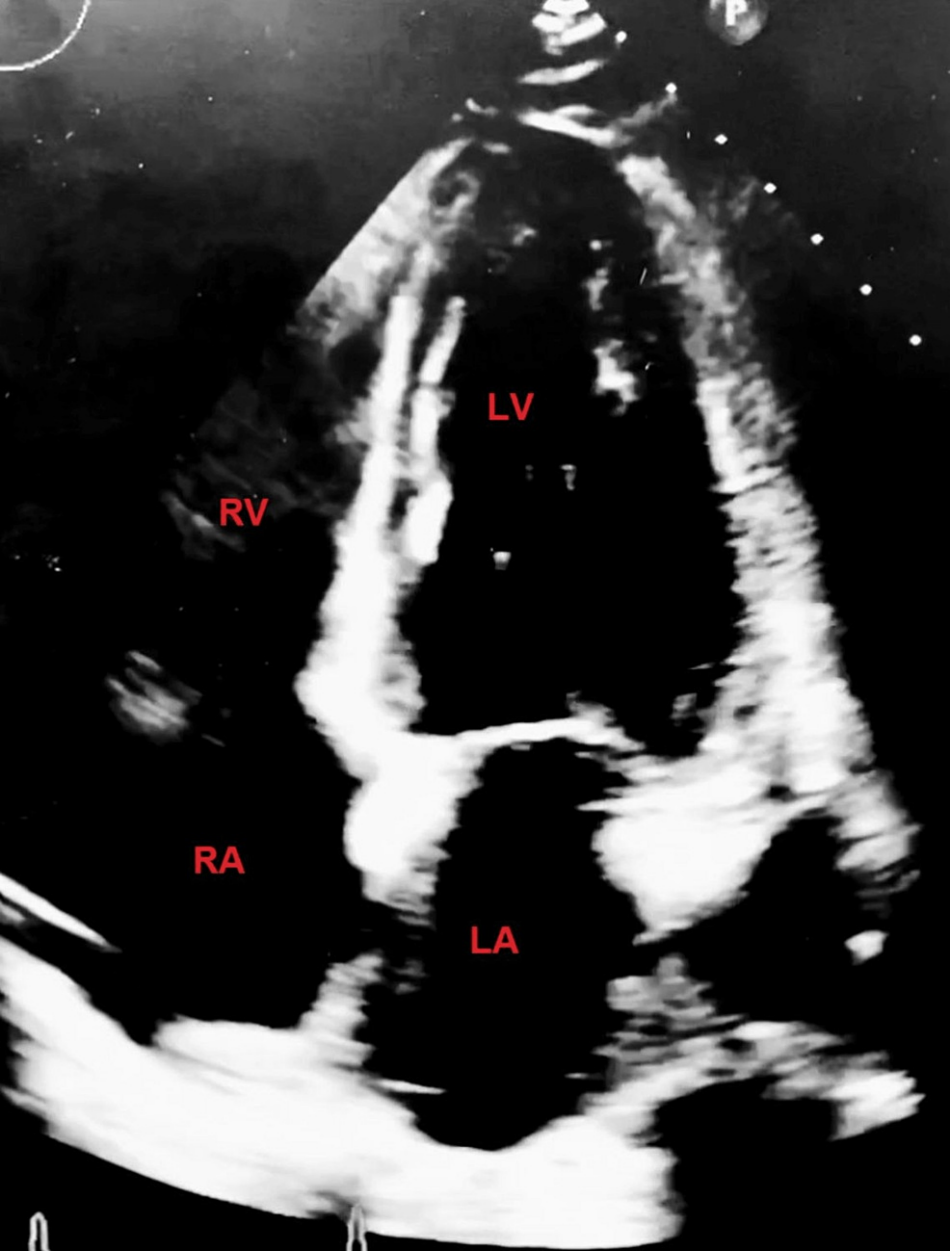
A two-day follow-up Echocardiogram demonstrated interval improvement in ejection fraction 43%. LV: Left Ventricle; LA: Left Atrium; RA: Right Atrium; RV: Right Ventricle

**Figure 4:**
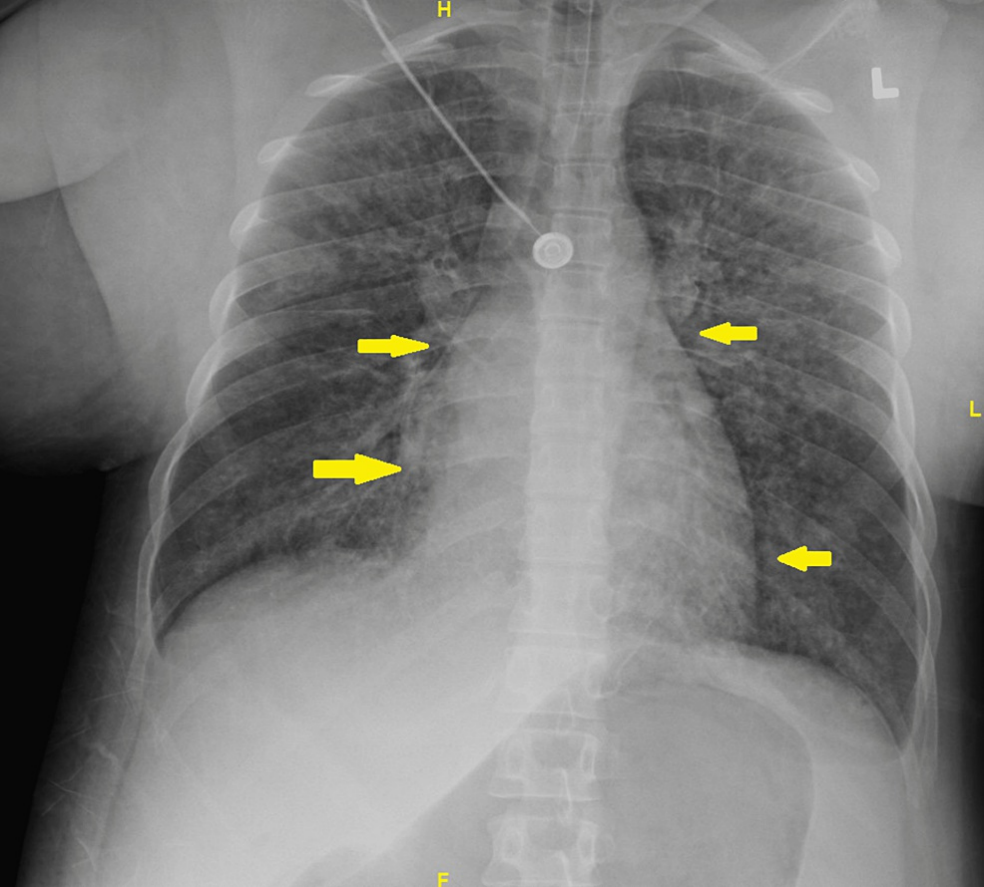
Portable chest X-ray performed in the immediate postoperative period demonstrating a cardiac silhouette in the upper limits of normal (yellow arrows). There is evidence of alveolar and interstitial edema and cephalization of vessels consistent with acute onset pulmonary edema.

**Figure 5:**
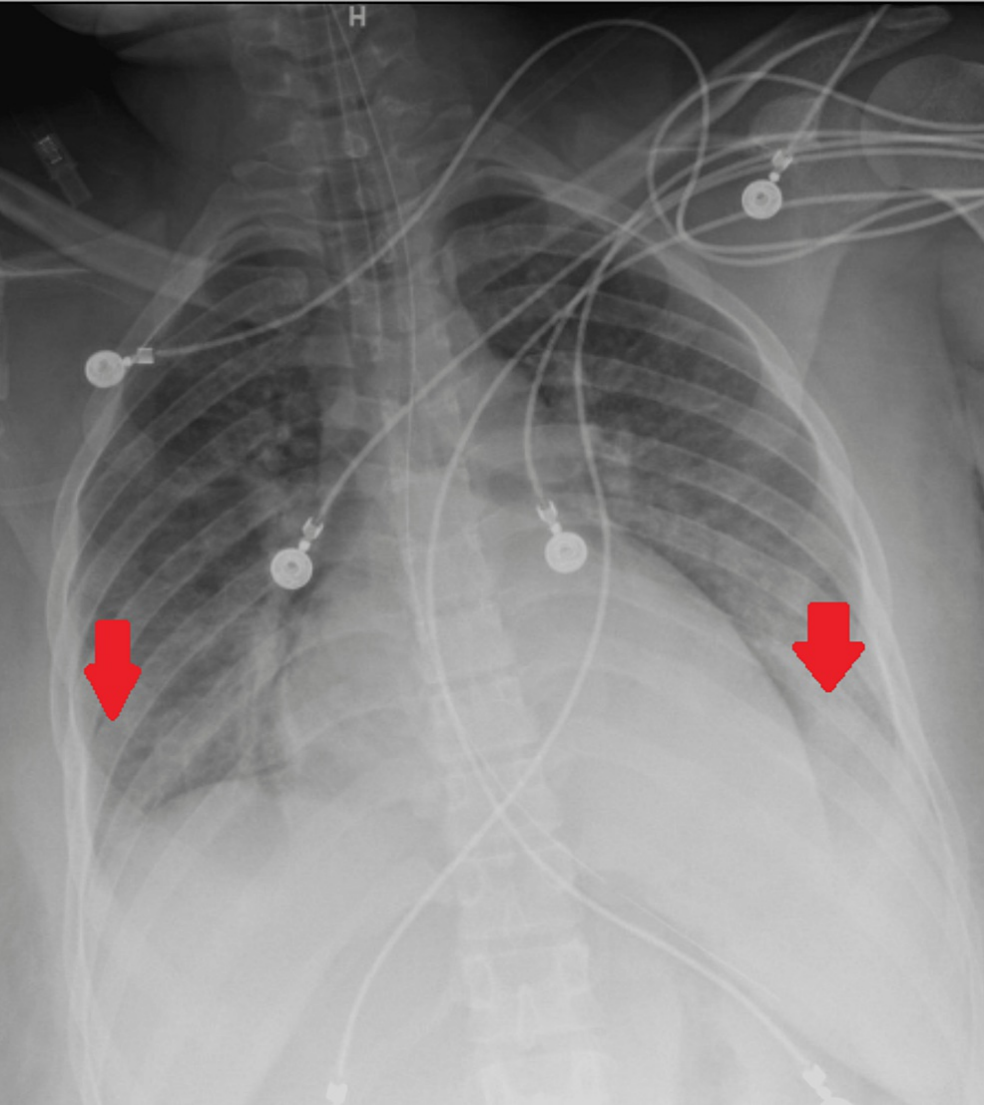
Serial chest X-rays performed in the following two days further demonstrated the development of bilateral pleural effusions (red arrows). Findings were suspicious for aspiration pneumonia.

**Figure 6:**
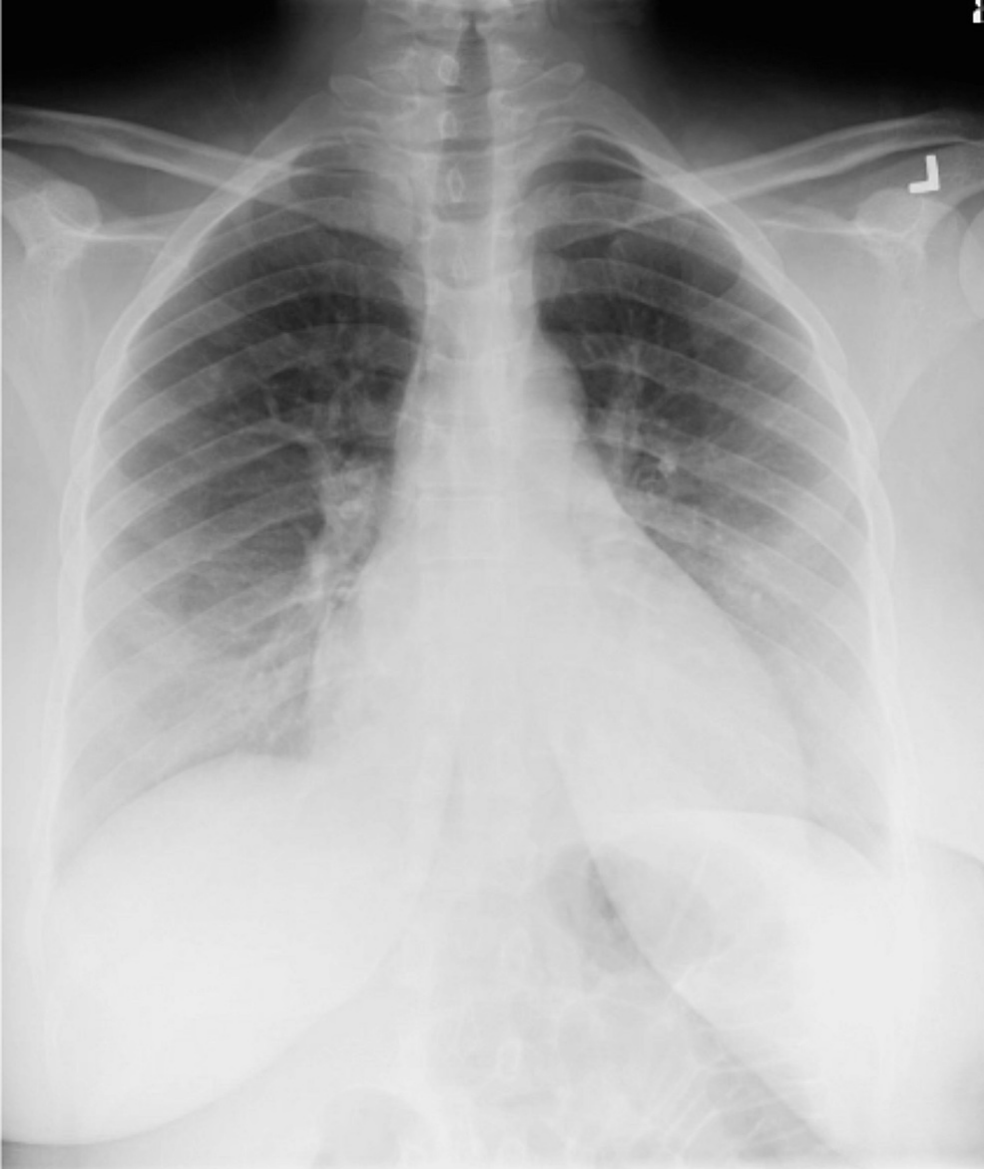
Chest X-ray with interval resolution of both bilateral pleural effusions and bilateral airspace opacities up on diuresis.

The patient’s preoperative electrocardiogram (Figure 1) demonstrated normal sinus rhythm, borderline low-voltage criteria, and no acute ischemia changes. Her immediate postoperative echocardiogram (Figure 2) revealed heart failure with a severely reduced left ventricular ejection fraction of 25%. Hyperkinesis of the apices of the left and right ventricles was demonstrated with hypokinetic mid-basal segments, notably seen with rTCM. Concentric left ventricular hypertrophy was noted. There was mild tricuspid regurgitation, elevated pulmonary arterial pressure, and a small pericardial effusion localized around the right ventricle. A two-day follow-up echocardiogram (Figure 3) demonstrated interval improvement in LV ejection fraction (LVEF) (43%). A portable chest X-ray performed in the immediate postoperative period showed mild cardiomegaly with evidence of alveolar and interstitial edema and cephalization of vessels consistent with acute onset pulmonary edema (Figure 4). Serial chest X-rays performed in the following two days further demonstrated the development of bilateral pleural effusions and findings suspicious for aspiration pneumonia (Figure 5). There was interval resolution of both bilateral pleural effusions and bilateral airspace opacities up on diuresis (Figure 6).

Initial laboratory studies upon admission have been summarised in Table 1. During admission, the patient’s WBC steadily decreased from 16.5 to 12.5 k/ul. She developed lactic acidosis type A, with her lactate trending from 1.1 to 1.9 on day 2 of admission before gradually stabilizing at 0.9 mmol/L. Her hemoglobin/hematocrit, platelets, and electrolytes remained stable and within normal limits.

**Table 1:** Laboratory values on admission

Lab Tests	Lab Values	Normal Range
WBC count	19.5 k/ul	4.8-10.8 k/uL
RBC Count	3.66 MIL/uL	4.50-5.90 MIL/uL
HGB	11.0 g/dl	12.0-16.0 g/dL
Hematocrit	32.8%	42-51%
Platelet	194 k/uL	150-400 k/uL
General Chemistry		
pH, arterial blood gas	7.399	7.350-7.450
pCO2, arterial blood gas	35.1 mmHg	35-45 mmHg
pO2, arterial blood gas	146 mmHg	83-108 mmHg
Sodium, Serum	138 mEq/L	135-145 mEq/L
Potassium, Serum	3.9 mEq/L	3.5-5.0 mEq/L
Blood Urea Nitrogen, Serum	7.0 mg/dL	8-26 mg/dL
Creatinine, Serum	0.6 mg/dL	0.5-1.5 mg/dL
Anion Gap, Serum	12 mmol/L	9-15 mmol/L
Bilirubin, Serum total	0.3 mg/dL	0.2-1.1 mg/dL
Bilirubin, Serum Direct Conjugated	0.2 mg/dL	0.0-0.3 mg/dL
Alkaline Phosphatase, Serum	150 unit/L	56-155 unit/L
Aspartate Transaminase, Serum	25 unit/L	9-48 unit/L
Alanine Aminotransferase, Serum	12 unit/L	5-40 unit/L
Lactic acid Level	1.1 mmoles/L	0.5-1.6 mmoles/L
Prothrombin Time	10 seconds	9.9-13.3 seconds
International Normalized Ratio	0.84	0.85-1.14
Serum Calcium	8.4 mg/dl	8.5-10.5 mg/dl
Pro BNP	5565 pg/ml	0-125 pg/ml
Troponin, Serum	28 ng/L	= 12 ng/L
Urinalysis	Bland, no proteinuria	
Urine Toxicology	Negative	

Given the temporal relationship of the patient’s symptoms, peripartum cardiomyopathy was initially suspected. However, upon the correlation of the echocardiogram findings with laboratory results, the patient was diagnosed with stress-induced cardiomyopathy, later determined to be rTCM. She responded well to 40mg intravenous furosemide twice a day, gradually improving cardiopulmonary symptoms. She was given ampicillin, clindamycin, and gentamycin for 24 hours postoperatively and later escalated. The patient was tapered off sedation with dexmedetomidine for weaning. The following morning, the patient was comfortable, responsive to commands, and denied any acute symptoms. She was extubated to BiPAP. The patient was then moved to a step-down unit and continued to demonstrate interval improvement in cardiopulmonary function.

The patient was discharged home eight days later with prescriptions for hydralazine, carvedilol, and isosorbide dinitrate and outpatient follow-up appointments with cardiology and pulmonology clinics. At her one-week follow-up, the patient and her baby were doing well.

## Discussion

Acute heart failure in the peripartum period has been reported in the literature since the early 1800s but was poorly understood and defined until a 1971 publication by Demakis et al. established the term: peripartum cardiomyopathy [1, 2]. The first significant attempt to categorize this condition was in a workshop convened in the 1990s by the US National Heart, Lung, and Blood Institute (NHLBI), which did so by defining it as heart failure that develops specifically either in the last month of pregnancy or in the first five months postpartum [2]. They defined inclusion criteria as LV systolic dysfunction with an LV ejection fraction (LVEF) of <45%, fractional shortening of <30%, or both [7, 8]. It is necessary to differentiate PPCM from other cardiac conditions which can present with heart failure [9]. Takotsubo cardiomyopathy (TCM) is a rare cardiac syndrome that was first described in Japan in the 1980s, presenting with temporary LV dysfunction, often induced by physical or emotional stress [2, 9]. Specifically, TCM is characterized by the ballooning of the apex of the LV following either of the stressors mentioned above and can often mimic acute myocardial infarction [3]. Historically, the predominant demographic TCM affected is postmenopausal women between 60-70 years old [1, 2]. Recently, the cases of young peripartum or postpartum women presenting with TCM have been rising [2]. Rarer still, rTCM is a variant in which the cardiac apex is hyperkinetic, and ballooning occurs at the heart base [3].

The etiology of PTCM is not well understood [2]. In a recent retrospective study of 37 patients without previous cardiac issues that presented with LV dysfunction in the peripartum period, the proportion of TCM, among other diagnoses of newly developed heart failure, was found to be greater than expected in Asian women [2]. Additionally, according to a systematic literature review comparing women after spontaneous delivery vs. women after cesarean delivery, women in the postpartum period after cesarean delivery were found to represent a higher risk of TCM. In these cases, the women diagnosed with TCM demonstrated LV systolic function normalization in 13.43 ± 10.96 days [4]. This is consistent with our understanding of TCM and its relatively transient nature compared to PPCM. The findings in the patient in the present study are also compatible with this finding, with a significant EF recovery and resolution of acute symptoms two days after admission.

In contrast to TCM, risk factors for PPCM include hypertensive disorders in pregnancy, maternal age >30 years, and Black race [6]. In a recent multicenter prospective study, Investigations of Pregnancy Associated Cardiomyopathy (IPAC), 100 women with PPCM were followed for 12 months [7]. This study found that EF recovery occurred for most women by six months, with minute changes after that. By 12 months, 71% of women recovered EF to >50%, with 13% reporting persistent cardiomyopathy with EF<35% [7]. TCM and PPCM are similar entities in the peripartum period and can be differentiated based on ECHO findings [2]. A retrospective observational study showed no significant differences in clinical characteristics between both, except PTCM had a higher incidence in multiparity. Also, the onset of symptoms was earlier in PTCM. Follow-up one-month and 12-month echo showed complete recovery of ejection fraction and a more favorable prognosis with PTCM than PPCM [10].

TCM and rTCM share a similar clinical presentation, including angina-like chest pain with or without dyspnea. Additional symptoms vary, including nausea, syncope, abdominal pain, and diaphoresis [1]. PTCM shares many similarities with PPCM. Both conditions present acute heart failure with decreased LVEF in young women with no previous cardiac issues. However, recovery of LVEF and long-term outcomes differ significantly [2]. In PPCM, outcomes and long-term ramifications are variable: patients may demonstrate complete recovery, long-lasting myocardial dysfunction, heart failure (HF), or rapid deterioration resulting in the need for durable mechanical support or cardiac transplantation [11]. Notably, because the diagnosis of PTCM was discovered relatively recently, it is possible that a significant number of cases of PTCM have been misdiagnosed as PPCM. Because it has been widely established that TCM usually affects postmenopausal women, PTCM has likely not been routinely considered in differentials. In contrast, PPCM is regarded as a diagnosis of exclusion [2].

The mechanism of TCM is poorly understood [2]. The various factors involved are microvascular dysfunction, catecholamine-induced cardiotoxicity, and complex neuroendocrine physiology, ultimately affecting the hypothalamic-pituitary-adrenal axis and cognitive centers of the brain [5]. The mechanisms proposed for rTCM are similar and include coronary artery spasm, coronary microvascular impairment, catecholamine cardiotoxicity, and estrogen deficiency [1]. The etiology of PPCM also remains poorly understood, but leading theories suggest that genetic predisposition, myocardial inflammation, and vascular dysfunction triggered by late-gestational maternal hormones may play a role [6, 7].

Due to the high proportion of TCM in postmenopausal women, reduced estrogen levels have been implicated as a leading contributing factor in TCM. In the female rat model, stress-induced apical ballooning is prevented by estrogen supplementation, corroborating this understanding. The cardioprotective role of estrogen has been long-established, with estrogen playing a key role in micro-circulation via endothelium-modulated mechanisms- and is implicated in coronary vasomotor abnormalities. Estrogen deficiency in menopause leads to endothelial dysfunction and sympathetic hyperactivity. In the postpartum period, rapid depletion of estrogen following the expulsion of the placenta could cause a similar constellation of downstream effects [2]. Additionally, the intense physical and emotional stress of delivery may lead to a surge of catecholamines, which have also been implicated as a leading hypothesized cause of TCM [1, 2].

TCM and rTCM often symptomatically mimic ischemic heart disease or acute coronary syndrome (ACS) [1]. In rTCM, electrocardiography changes, including ST-segment elevation, T wave inversion, newfound bundle branch block, or prolonged QT interval, can all be observed [1]. LV wall abnormalities are classic findings, with hypokinesis or dyskinesis of the LV apex, which usually only affect one coronary artery territory [1, 2]. Cardiac troponin is typically expected to be modestly elevated, along with a high brain natriuretic peptide (BNP) [1]. The typical workup for suspected ACS should be revelatory for a diagnosis of rTCM. This should include blood sample analysis, electrocardiogram, transthoracic echocardiogram, and coronary angiogram [1]. Ventriculogram or cardiac magnetic resonance imaging can also be considered. Cardiac catheterization often yields a definitive diagnosis during left ventriculography [1].

Specific guidelines for the treatment of rPTCM have not yet been established [1]. However, the treatment guidelines for rTCM have been established by the American College of Cardiology (ACC), and the treatment is primarily supportive with additional management of complications. The treatment guidelines for TCM are similar, though the reversed anatomy may require special consideration with management [1]. Because TCM often mimics ACS, ACS guidelines are typically followed for both TCM and rTCM. This includes aggressive hemodynamic and pharmacologic support to reverse the decline in LV function rapidly [1].

Additionally, in patients with dynamic LV obstruction, beta blockers can be cautiously administered to reduce the contractility of the affected segment of the myocardium. If present, pulmonary edema can be treated with upright posture, oxygen, and diuresis [1]. In contrast, PPCM treatment focuses on volume control, mitigating negative neurohormonal response, preventing life-threatening arrhythmias, and preventing thromboembolic complications [12]. Bromocriptine therapy has been proposed as a treatment option and has shown conservatively promising results. The latest study supporting bromocriptine therapy was a multicenter trial with 63 patients with PPCM, where bromocriptine treatment was associated with an increased rate of LV function recovery [12].

We need more randomized controlled trials for medical therapies for Takotsubo cardiomyopathy (broken heart syndrome). A meta-analysis done by Singh et al. showed that angiotensin receptor blockers and angiotensin-converting enzyme blockers might decrease the recurrence of stress-induced cardiomyopathy [13]. Novel therapies for TCM are under investigation, including the use of estrogen in animal models, routine dosing of an endothelin antagonist and adenosine, and long-term beta-blocker treatment which has been theorized to reduce the likelihood of recurrence [1].

## Conclusions

Physicians should be aware of the growing number of cases of the reverse variant of Takotsubo cardiomyopathy among peripartum women. Echocardiogram in these patients is characterized by hyperkinetic cardiac apex with ballooning of the heart base. Treatment of these patients is primarily supportive - oxygen supplementation and diuresis have been useful for symptoms of pulmonary edema, and beta blockers have been shown to reduce contractility of affected segments where the concern of dynamic left ventricle obstruction exists.
